# A blended learning training programme for health information providers to enhance implementation of the *Guideline Evidence-based Health Information*: development and qualitative pilot study

**DOI:** 10.1186/s12909-020-1966-3

**Published:** 2020-03-18

**Authors:** Jana Hinneburg, Julia Lühnen, Anke Steckelberg, Birte Berger-Höger

**Affiliations:** Institute for Health and Nursing Science, Medical Faculty, Martin Luther University Halle-Wittenberg, Magdeburger Str. 8, 06112 Halle (Saale), Germany

**Keywords:** Training, Health information, Evidence-based medicine, Guideline implementation, Guideline adherence

## Abstract

**Background:**

The *Guideline Evidence-based Health Information* was published in 2017 and addresses health information providers. The long-term goal of the guideline is to improve the quality of health information. Evidence-based health information represents a prerequisite for informed decision-making. Health information providers lack competences in evidence-based medicine. Therefore, our aim was to develop and pilot-test a blended learning training programme for health information providers to enhance application of the guideline.

**Methods:**

1. Development:

We developed the training programme according to the Medical Research Council guidance for developing and evaluating complex interventions. The training programme was planned on the basis of problem-based learning. It aims to impart competences in evidence-based medicine. Furthermore, it comprises the application of criteria for evidence-based health information.

2. Pilot testing:

We conducted a qualitative pilot study focusing on the acceptability and feasibility of the training programme. Health information providers were recruited and in-house training sessions were offered.

Feasibility and acceptability were explored by structured class observations and in semi-structured focus group interviews with the participants after the training sessions. The transcripts and documentations were analysed using qualitative content analysis according to Mayring. The training was revised iteratively according to the results.

**Results:**

We conducted two training courses with 17 participants between November 2018 and March 2019. The adequacy of the training for the target group was identified as a major issue. There was significant heterogeneity concerning previous knowledge. Some wished to delve deeper while others seemed to be overwhelmed. In general, the work tasks were understandable. However, the participants asked for a more detailed theoretical introduction in advance. The practical relevance of the evidence-based medicine contents was rated rather low compared to the content about evidence-based health information. Based on these results, we revised the programme.

**Conclusions:**

Overall, the training proved to be feasible for implementation. Meeting the needs of all the participants was a challenge, since they were heterogeneous. Not all of them will be able or intend to implement the training contents into their working routine to the full extent. The implementation will be evaluated in a randomised controlled trial.

## Background

Evidence-based health information (EBHI) is a prerequisite for informed decision-making, which is based on adequate knowledge and implies decisions which are congruent with peoples’ preferences and values [1]. Most German citizens prefer shared decision-making [2]. The German Patients’ Rights Act underlines the right to comprehensive and comprehensible information and implies patient participation in medical decisions [3]. Furthermore, the German National Cancer Plan defined shared decision-making as one of the planned goals [4].

There is a flood of health information available on the internet, but most of the information does not fulfil the quality criteria for good health information. Unfortunately, quality criteria are not considered in the ranking algorithms of the search engines so that the search for high-quality health information is challenging. In addition, people often rate familiar and commercial online information sources as being trustworthy [5].

In Germany, good practice guidelines for health information have been published by a working group in the German Network for Evidence-based Medicine. They provide support for authors and publishers of EBHI by offering quality criteria [6]. Even though the criteria for EBHI have long been defined [7, 8], implementation into practice is lacking [9, 10]. Interviews with providers of health information revealed shortcomings regarding their competences in evidence-based medicine (EBM) [11].

In 2017, the *Guideline Evidence-based Health Information* was published by the German Network for Evidence-based Medicine [12]. It addresses providers of health information and aims to improve the quality of health information by giving 21 recommendations on EBHI based on systematic evidence syntheses. The guideline includes evidence-based recommendations for the development, content and presentation of EBHI (e.g. numerical and graphical representation). Moreover, it comprises methodological and ethical requirements like the development process, contents of EBHI and target group involvement.

Several strategies for implementing guidelines have been discussed. There is comprehensive evidence that the implementation of guidelines in combination with a training programme can improve implementation [13]. In contrast to medical guidelines, the *Guideline Evidence-based Health Information* requires methodological competence to systematically search and appraise the evidence before it can be presented according to the guideline recommendations. Therefore, this implementation strategy seemed to be essential for the guideline*.*

This qualitative study describes the development and piloting of a blended learning training programme for health information providers to enhance implementation of the *Guideline Evidence-based Health Information*. It is part of a project on the implementation of the guideline in combination with a training programme which will be evaluated in a randomised controlled trial registered on the ISRCTN registry (ISRCTN96941060). The study protocol of the randomised controlled trial has been submitted to *Trials*. The aim of this study was to explore the feasibility and acceptability of the programme. The study protocol is available online [14].

## Methods

We followed the Medical Research Council guidance for developing and evaluating complex interventions with focus on acceptability and feasibility (phase I and II) [15]. The results are reported according to the revised *Criteria for Reporting the Development and Evaluation of Complex Interventions in healthcare* (CReDECI 2) [16] and *COnsolidated criteria for REporting Qualitative research* (COREQ) (see Additional file 1) [17].

### Development of the training programme

We developed the training programme following Kern’s six-step approach for curriculum development for medical education [18]:

- *Step 1: Problem identification and general needs assessment*

The paucity of EBHI as a prerequisite for participation and informed decision-making has repeatedly been described. The *Guideline Evidence-based Health Information* summarised this background and was therefore used as the main source to derive the general needs.

- *Step 2: Targeted needs assessment*

Health information providers, who develop and publish health information, have been defined as the target group, which is not limited to a special profession or level of education. However, we expected that most of the participants would be health professionals with an academic background. Competences required for developing EBHI were defined and squared with the results of exploratory interviews previously conducted with the target group in order to set the scope of the training [11].

- *Step 3: Goals and objectives*

The teaching goals were inspired by the basic curriculum for evidence-based decision-making of the German Network for Evidence-based Medicine [19] and formulated accordingly. They are broadly defined in Table 1.

- *Step 4: Educational strategies*

**Table 1:** Teaching goals

Module	Goals
Module 1: EBM training	
1.1 Introduction to EBHI	• Participants gain an overview of the development process of EBHI and reflect on their own practice. • Participants start to consider EBHI as the prerequisite for informed decision-making.
1.2 Treatment studies	• Participants understand the difference between association and causality and that randomised controlled trials (RCTs) are designed to establish a causal relationship. • Participants know the characteristics of RCTs. • Participants are able to interpret the results of RCTs and critically appraise them.
1.3 Evidence syntheses	• Participants are able to interpret the results and critically appraise systematic reviews and meta-analyses. • Participants describe the development process of guidelines and are aware of their limitations.
1.4 Diagnostic studies	• Participants are able to identify the major study designs for diagnostic studies. • Participants are able to calculate and interpret test accuracy. • Participants recognise the problem of overdiagnosis and overtherapy.
1.5 Systematic literature search	• Participants are able to conduct systematic literature searches to identify literature appropriate to their research question.
Module 2: Application of the guideline	
	• Participants are able to develop EBHI and document the development process. • Participants know about and apply strategies for piloting EBHI. • Participants consider EBHI as the prerequisite for informed decision-making.

The educational strategies were developed using information gained in steps 1–3. The training programme was planned on the basis of problem-based learning to foster active learning. Problem-based learning was chosen since it complies with the paradigm of EBM such as critical thinking. A case example about smoking cessation was set up as a practical challenge. Problem-based learning is intended to increase knowledge and understanding by using appropriate problems that serve as a stimulus for learning [20]. Due to the limited amount of time, the format of problem-based learning was shortened: The problem and teaching goals were formulated by the teachers instead of offering a complete open setting. The problem of smoking cessation was chosen to enable providers to understand the relevance of scientific knowledge [20] in the development process of health information and also to promote intrinsic interest and motivation. The topic comprises evidence-based methods for smoking cessation, for instance counselling, medications (e.g. bupropion) and nicotine replacement therapy.

- *Step 5: Implementation*

This step corresponds to the piloting of the training programme.

- *Step 6: Evaluation and feedback*

The training programme was revised according to the results of this qualitative pilot study and the implementation will be evaluated in a randomised controlled trial.

**Fig. 1:**
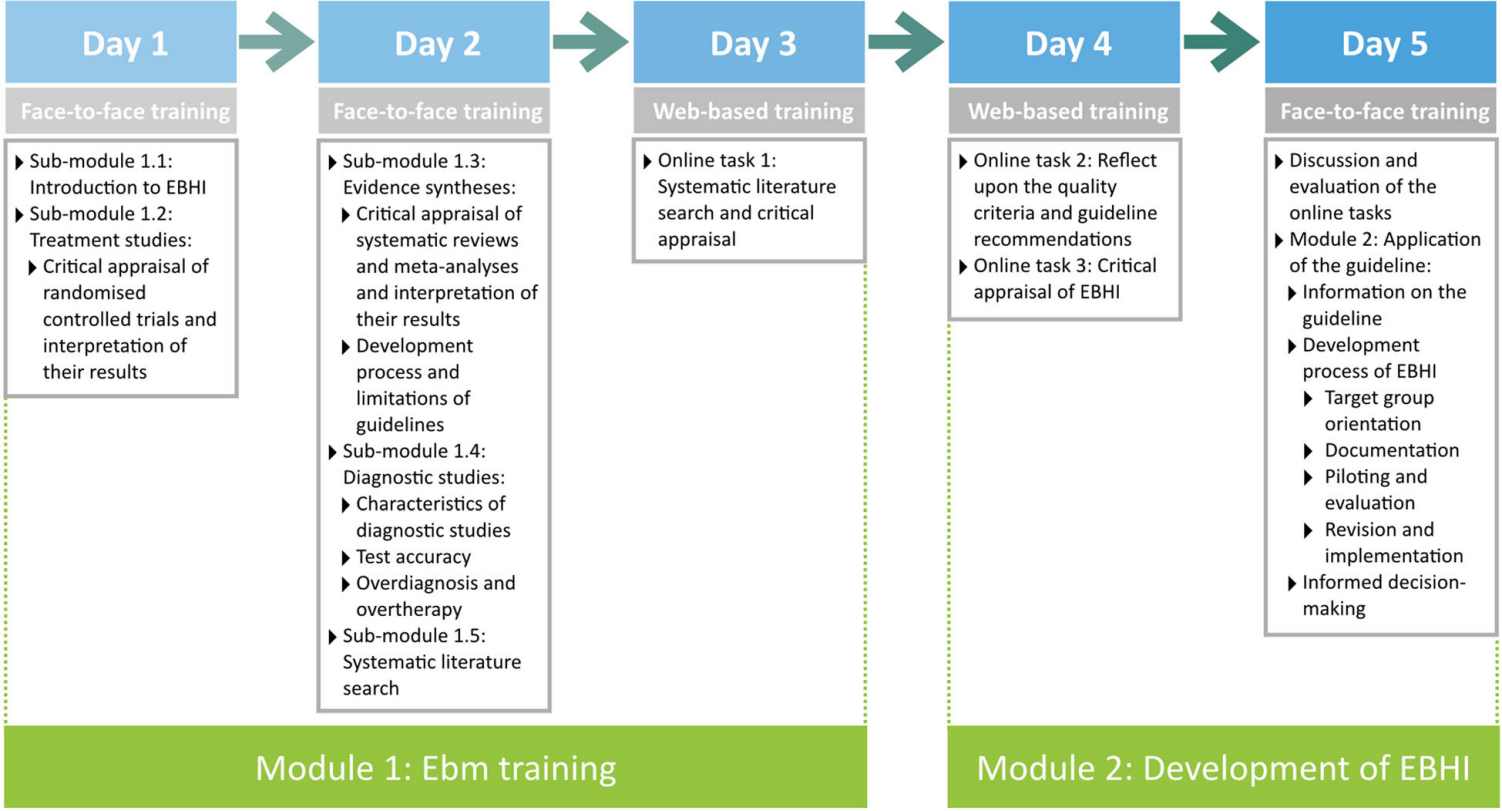
Modular structure and contents of the blended learning training programme

The training programme consists of two modules (Fig. 1). The first module comprises EBM training (sub-module 1.1–1.5) and aims to impart competences in searching for, critically appraising and extracting relevant literature according to the principles of EBM. The second module is about the application of the guideline and comprises the criteria for EBHI, critical appraisal of health information and the reflection of providers’ processes for developing health information. A folder containing the training materials, sorted according to the modules, was provided for the participants.

The training programme was designed in a blended learning format. The first module comprises 2 days of face-to-face training followed by 1 day of web-based training (approximately 8 h per day). The second module is designed as an inverted (or flipped) classroom scenario (participants deal with the learning material prior to the face-to-face training) [21]: 1 day of web-based training followed by 1 day of face-to-face training.

The length of the online phase was coordinated with the participating institutions. A two-week online phase was recommended so that the participants would have enough time to complete the tasks. For the web-based training, the learning management system ILIAS was used. ILIAS included slideshows and text resources combined with online tasks and video tutorials. In addition, further information on the course contents was provided so that the participants could deepen the contents of the face-to-face phase. They were encouraged to upload the results of their online tasks and to receive feedback during the face-to-face training.

### Piloting and feasibility of the training programme

We conducted a qualitative pilot study with focus on acceptability and feasibility of the training programme.

#### Setting and sample

The recruitment of health information providers was performed on an institutional level. Participating institutions were recruited via already existing contacts with the institutions. The directors of the institutions were contacted directly by telephone and asked if they were interested. We offered in-house training sessions for those employees involved in the development process of health information. There were no further requirements for participation. The teaching programme was designed for a maximum of 15 participants per course.

#### Data collection and procedure

Health information providers participated voluntarily in the training programme. An information sheet was sent out to the participants via email prior to the training. Written informed consent was obtained at the beginning of the training and data collection was conducted during the training sessions. Training and data collection were carried out by BBH and JH, who gave a brief introduction to their research. Baseline characteristics of the participants, including sex, age, education status, self-estimated English skills, self-estimated EBM knowledge and their qualifications for the development of health information, were assessed.

Feasibility and acceptability of the training programme were investigated from the perspectives of both learners and teachers. Acceptability was defined as the acceptance of teaching methods at classroom level and the relevance of contents at the practical level. Feasibility was defined as the practicability and usability of the training programme and its contents. At classroom level the focus was on the following aspects: comprehensibility of the learning and teaching materials and contents, structure of the training, scheduling, usability of the web-based learning environment and target group orientation. Potential application barriers, framework conditions and motivation were assessed at the practical level.

Furthermore, structured class observations were carried out by at least one silent observer taking field notes. Main foci of the observations were the reactions of the participants to teaching methods, the comprehensibility of materials and content, the interaction between teachers and participants (e.g. questions from the participants), problem-solving in the classroom situation as well as scheduling. The teachers also took fields notes, which were discussed with observers afterwards. Work products such as flip charts, processes and interactions were documented.

After module 1 and module 2, feasibility and acceptability were explored from the personal perspective of the participants in semi-structured focus group interviews. The participants and three researchers (BBH, JH and AS), who led the focus groups, were present. At least one of these researchers had advanced experience in conducting focus groups. The researchers followed an adapted semi-structured interview guide which had been developed in a prior pilot study [22] to cover pre-defined categories but also allowed new categories to come up for discussion (see Additional file 2). Field notes were taken additionally during the focus group interviews which were also audio-recorded and transcribed verbatim. A short feedback (flash light) by the participants followed the online phase. The transcripts and findings were not returned to the participants for comment and/or correction because this would have involved a considerable additional organisational expense.

#### Data analysis

Analyses of the baseline characteristics were descriptive. The transcripts and documentations were analysed using qualitative content analysis according to Mayring [23]. The data were combined using between-methods triangulation [24]. BBH and JH coded the transcripts applying a coding guideline (see Additional file 3) and using the software QCAmap [25]. Initially, a category system was deductively derived from the research questions. Despite the already existing category system, categories could be adapted flexibly via feedback loops during data analysis. During the coding process, categories were adapted and subcategories were inductively derived from the data. Afterwards, two researchers discussed the results. Theoretical data saturation was intended by an iterative process of testing, analysing and revising the training programme.

## Results

### Participants

We performed two pilot courses with employees from a health insurance company (*n* = 5) and a health information provider (*n* = 12) between November 2018 and March 2019. All the participants completed the training programme. The mean age of the participants was 41 years (range 28–51) and nine of 17 participants were female. Fifteen of 17 participants had a university degree (one in medicine) and two had a general education school-leaving certificate. The participants rated their English skills as being elementary (A2) (*n* = 2), intermediate (B1) (*n* = 2), upper intermediate (B2) (*n* = 10) or as being advanced (C1) (*n* = 3). They perceived their EBM knowledge as little (*n* = 2), moderate (*n* = 11), good (*n* = 3) and very good (*n* = 1). They all reported that they had acquired their qualification for the development of health information through their occupational activity (“learning by doing”) and by individual study using literature. The participants stated that they used the following sources for the development of health information: medical databases (*n* = 9), journals (*n* = 10), guidelines (*n* = 12), experts (*n* = 11) and others such as diverse online sources and conferences (*n* = 7).

### Feasibility and acceptability

After the second training course, theoretical data saturation was assumed since few or no new insights were revealed. The focus groups lasted approximately 30 min. All the participants had the opportunity to express themselves and contributed to the focus group. Seven categories were established via qualitative content analysis: 1. expectations and motivation, 2. framework conditions of the training, 3. interaction and teaching methods, 4. planning of the training programme, 5. value and design of the learning and teaching materials, 6. comprehensibility of the contents and 7. practical relevance and feasibility. The field notes of the class observations and the results of the focus group interviews were triangulated. Most of the results from the class observations were congruent with those of the focus groups. Additionally, usage data (number and duration of accesses) from the learning management system ILIAS were considered. Quotes were translated verbatim.

#### 1. Expectations and motivation

This category describes the participants’ main motives for taking part in the training and what they expected of it.

In the first pilot course, the participants regarded the potential competitive advantage, unique selling point and their strategic development as the motivation for the training. Moreover, they saw it as an opportunity to have an improved argumentation basis for patients demanding services which are not covered by their health insurance, for example individual health services.

The participants’ expectations of the second pilot course differed somewhat from those of the first pilot course. This was mainly due to the fact that the participating institution’s main purpose is to develop health information. They mentioned that they wanted to get an overview of the complex market of health information. Concerning health information, they were interested in learning about do’s and don’ts and how to translate evidence into plain language. Furthermore, they wanted to acquire EBM knowledge as well as statistical literacy, because they were afraid of making mistakes.

The participants described an area of tension between the criteria of EBHI and practice in both pilot courses. One participant was concerned that patients could be overwhelmed by EBHI, perhaps because they might not want to make a decision on their own.

#### 2. Framework conditions of the training

This category includes the adequacy of the training for the target group, the adequacy of the time frame and the technical realisation of the online phase.

The adequacy of the training for the target group seemed to be a major topic in the focus group interviews. The participants were not sure about the definition of the target group and whether or not they belonged to it. On the one hand, this was the case because some participants did not identify or classify themselves as developers of health information since it was not their main task at work. On the other hand, there was significant heterogeneity among participants. Some were already trained in EBM and others were novices. One participant stated:

“*I think it was bit by bit, actually. One was gradually introduced. I’m a beginner. Not from the EBM department. Therefore, I found it to be a good introduction to the topic*.” (Focus group 2)

But then again, one participant expressed her concern since she was not able to follow the contents:

“*You expect previous knowledge that I don’t have. And if you are always lagging behind a bit, it becomes difficult*.” (Focus group 1)

This implied that novices would not attain a deeper understanding. Other participants explained that they could follow quite well. It became clear that some participants with previous knowledge would have wished to delve deeper into a few of the topics and for others the contents seemed to be overwhelming:

“*Surprisingly, lots of things weren’t new for me. Therefore, I could follow quite well. I’ve done an EBM training before and I’m working in this field. Nevertheless, there were some new aspects and things one starts to see differently. I wasn’t bored. Of course, I would have delved deeper into some aspects in another group. But it was clear that those who had just started absolutely could not… That’s of course the challenge in such a diverse group*.” (Focus group 2)

The class observations coincided with the results of the focus groups. Especially in the second pilot course during the sub-module *Treatment studies* it became obvious that some participants already had knowledge of statistical terms, whereas others needed extensive explanations with the help of practical examples.

The participants explained that the heterogeneity of the target group fostered mutual understanding of the different departments within one institution. One participant said:

*“I liked the fact that so many out of different departments participated. Because sometimes I’ve the feeling that they don’t really know what we’re doing and smile about it. Why we need so much time and come forward with quality and this and that. Insofar, I think it’s good for the whole team. […] Although it was heterogenic, it was productive.”* (Focus group 2)

Nevertheless, some participants suggested dividing up the training into different smaller modules or different courses depending on the prior knowledge:

“*I sometimes asked myself the question, who the target group is. One would have to break up the training into smaller modules. […] For different target groups so that it is possible to focus on different levels of knowledge*.” (Focus group 2)

However, one participant mentioned that it would not be possible to disentangle the target group.

Some participants appreciated the compact format of the training, but it was discussed whether the scope of the training should be modified, depending on the previous knowledge of the target group. It was mentioned that the training programme requires quite a large amount of time and staff resources by the participating institutions.

Moreover, the participants gave feedback on the learning management system ILIAS. They said that it could not be operated intuitively. In addition, minor navigation problems in ILIAS were reported (e.g. locating download material). The usage data of ILIAS showed that all of the participants visited the platform. The duration of use differed significantly, partly because the participants organised themselves in groups to complete the online tasks. Additionally, some of the participants used the platform only for up- and downloading the online tasks.

#### 3. Interaction and teaching methods

This category characterises the exchange between the involved people as well as the adequacy, realisation and acceptance of the teaching methods.

The participants described the learning atmosphere as pleasant and open for questions. The interactive instructional design, feedback and explanations were appreciated. Some participants said that the exercises and work tasks enhanced the learning effect:

*“The practical exercises were very helpful to get a deeper understanding.”* (Focus group 1)

Some of the working phases were perceived as too long as they required increased attention. Group work was judged to be well suited for the work task on systematic literature search (e.g. development of the PICO scheme).

#### 4. Planning of the training programme

This category describes the adequacy of the planning of the training programme.

Some of the participants found it difficult to integrate the online phase into their working routine. One participant described the online phase and work task on systematic literature search as follows:

“*I think I’ve done everything. But I’m much faster than my colleagues who’ve never done that before. And some of us had completed the first work task in a large group. […] Self-organisation isn’t as easy sometimes. But we did a quite good job*.” (Focus group 2)

Furthermore, it was mentioned that the online task on systematic literature search requires more time, especially for beginners. Implementing comprehensive literature searches into the working routine seemed to be challenging.

Sometimes, a common thread was missing and the training concept was not transparent for some participants:

“*What was missing… The big picture of the whole training concept. What are we doing how, and what is building on what and where can I expect what. Because some things will be part of the third attendance day, and I don’t know, will they be a topic or do I have to ask*.” (Focus group 2)

Moreover, especially the participants of the first course wished for a longer input phase before performing the work tasks. This was particularly the case for the sub-module *Treatment studies*:

“*It would have been easier for me if we had dealt with the terminology and the question ‘where do I have to look’ first and then with the tasks. Because those are fun and interesting, and I really want to know it. And then it’s frustrating to do the task like chewing a hard piece of meat and afterwards you get the tenderiser*.” (Focus group 1)

The class observation also revealed that a common thread was missing in this sub-module and that several topics seemed to arise at random.

#### 5. Value and design of the learning and teaching materials

This category explains how the participants rated the learning and teaching materials with regard to their practical relevance and design.

The training folder and printed presentations were appreciated for making notes and repeating the contents. Some participants would have liked digital slides as well. In general, the learning material was rated as clear and readily understandable. The authenticity of the studies used in the work tasks was emphasised positively:

“*I liked the real texts. The use of real studies. That’s very concrete. That’s the real work and the right material to work on. It’s irritating if it [the studies in the work tasks] looks completely different*.” (Focus group 2)

Additionally, the checklist for appraising health information was judged to be very helpful:

“*I found the checklist for appraising health information very helpful for reflecting upon my own work*.” (Focus group 2)

#### 6. Comprehensibility of the contents

This category means the comprehensibility of the work tasks, terminology, criteria of EBHI and includes suggestions for additional contents.

The work tasks were judged to be understandable but some of them were also extensive and challenging. On the one hand, some participants considered the translated studies as too long for reading. On the other hand, some of the more experienced participants suggested that they could be offered the original English language studies. Critical appraisal of the studies was regarded as too demanding in the first pilot study. Some wished for a better explanation of the procedure of a study and a short guideline on how to read a study.

Furthermore, the calculation of absolute numbers from a meta-analysis was perceived as difficult. Many of the participants asked for definitions of (statistical) terms (e.g. *p* value and confidence interval) combined with practical examples before attempting the work tasks.

The participants gave some suggestions for additional contents. Since different departments of the institutions participated in the second pilot study, they had different perspectives on the development of health information. Therefore, they wanted to work on the interface between EBM and communication:

“*I think it’s important to discuss the interface between communication topics and EBM*.” (Focus group 2)

One participant suggested that it could be helpful to appraise health information from the participating institutions:

“*I would have found it exciting to take a piece of our information and discuss what is already good and what isn’t so that we are able to test the results concretely*.” (Focus group 2)

Moreover, some participants missed a further explanation of the evidence behind the guideline recommendations as well as the evidence concerning pictures, comics and multimedia formats. Regarding the criteria of EBHI, some participants wanted to intensify the practical application of the criteria.

“*Of course, I would have liked more about how to apply it since I already know a lot of it from EBM. Extend this process of reflection, which we’ve worked on today*.” (Focus group 2)

They found it interesting that there might not be a clear answer to some questions concerning the development of EBHI due to insufficient evidence. The definition of the target group and goal of an EBHI were identified as crucial criteria:

“*Especially the goal and target group, that has to be focused very clearly before developing information or it has to be taken into consideration. Those are aspects that weren’t clear to me before. Even if I’ve read it. I completely lost sight of it. Because it’s logical that we do it to…? But why are we doing it? I found it quite interesting*.” (Focus group 2)

Furthermore, it was discussed whether it is ethical to leave out information that, for instance, does not have a good evidence base.

#### 7. Practical relevance and feasibility

This category describes the practical relevance and applicability in practice.

Systematic literature search was regarded as interesting and informative. The sub-module *Diagnostic studies* was described as exciting as well. Reading the studies promoted critical thinking. Especially the explanation of different features of a meta-analysis seemed to be very helpful.

The case example about smoking cessation was considered to be relevant and helpful.

“*The example and the topic were well chosen because it’s a topic everyone is interested in and that everyone knows*.” (Focus group 1)

One participant described the case example as too abstract. The continuity of the example was considered as positive as was the fact that it caused empathy and structured the training:

“*I liked Ms Lemke because one can put oneself in the position of a real person. She was present all the time and structured it for me. I liked this approach*.” (Focus group 2)

Moreover, the case example induced the reflection of the target group definition. In this case, the case example (Ms Lemke) suggested a need for information.

The practical relevance of the EBM module was rated rather low compared to the second module. The second module was described as more comprehensible and practical:

“*It was much more comprehensible today than the two days before. This was mainly because of the practical examples, which are more familiar to me from daily business. I liked it really a lot*.” (Focus group 1)

In general, the participants saw the training as a good opportunity to deepen specific topics and many of them affirmed that they profited from it. However, the scientific character required a comprehensive training. Furthermore, it encouraged respect concerning the development of EBHI:

“*Would I dare to develop an EBHI based on those three days of training? No way. I have to say so. I’ve great respect. But I think one sees those things with different eyes. And details are revealed, critical aspects, and that helps*.” (Focus group 1)

Regarding the necessary resources in the workplace, it was mentioned that it is an advantage if there are several departments developing health information together. Additionally, the participants named time as the most relevant resource.

### Revision

The training programme was revised iteratively based on our results. However, most of the changes were minor ones on slides or in the work tasks in order to improve clarity. Table 2 shows the identified need for revision and the revision conducted.

**Table 2:** Results of the focus groups, class observations and revision process

Identified need for revision	Revision conducted
Focus groups and class observations: Whole training programme: A common thread was missing sometimes and the training concept was not transparent for some of the participants.	The explanation of the training programme’s structure had already been included in the sub-module 1.1 *Introduction to EBHI* and in the training folders. The structure will be made permanently visible in following training sessions (e.g. by using a poster).
Focus groups and class observations: Sub-module 1.2 *Treatment studies*: The participants asked for a theoretical introduction to statistical and methodological terms on the basis of practical examples in preparation for the work tasks.	The module was better structured and an input phase including practical examples was planned before the work tasks after the first pilot study. Additional practical examples were added after the second pilot course.
Focus groups and class observations: Sub-module 1.3 *Evidence syntheses*: Critical appraisal of the studies was regarded as too demanding in the first pilot study.	The work task on critical appraisal of a systematic review was divided up and planned as a group task.
Focus groups and class observations: Sub-module 1.2 *Treatment studies* and 1.3 *Evidence syntheses*: Reading the translated studies was considered as very challenging and time-consuming by the participants of the first pilot study.	The study texts were shortened by deleting less meaningful passages.
Focus groups: Online phase: Some participants mentioned that the online task on a systematic literature search requires more time, especially for beginners. The implementation of comprehensive literature searches into the working routine seemed to be challenging.	The work task was defined as optional for participants who are not involved in the methodical development of health information.
Focus groups and class observations: Module 2 *Application of the guideline*: Some participants requested further explanation of the evidence behind the guideline recommendations as well as the evidence concerning pictures, comics and multimedia formats.	Some slides containing the evidence behind relevant recommendations were added.

## Discussion

Overall, the training was well accepted and it proved to be feasible for implementation. The extensive EBM knowledge encouraged a deeper understanding of the complex development process of EBHI. However, it led to the fact that some participants felt overwhelmed by the contents and did not see the necessity of learning the extensive EBM contents.

The adequacy of the training for the target group seems to be a major issue since the participants in the pilot training sessions were heterogeneous regarding their prior knowledge and their involvement in the process of developing health information within their institutions. Not all of them will be able to or intend to implement the training contents into their working routine. Nevertheless, the training encouraged respect concerning the development of EBHI because the participants realised that developing EBHI is a complex process which requires comprehensive skills, is time-consuming and staff-intensive. The adequacy of the training for the target group will probably remain a challenge since there is no defined qualification for developing EBHI. Nevertheless, the pilot study showed that an interprofessional training seems to be an opportunity since different professions appeared to profit from the perspectives and opinions of others. A lack of English skills might be a problem in the process of developing EBHI. English skills are necessary to search for, critically appraise and extract relevant literature according to the principles of EBM. We offered the participants the translated studies but in practice this will not be the case.

To our knowledge, until now this training of health information providers for developing EBHI is unique. With regard to the EBM contents, some of the results of this pilot study are similar to those of another training course conducted for physicians and medical students to enhance their competences in evidence-based decision-making [26]. Here too, the participants asked for a theoretical introduction to statistical and methodological terms on the basis of practical examples. Studies revealed shortcomings regarding medical professionals’ statistical literacy [27–30]. For instance, Jenny et al. (2018) conclude that medical students and professionals should receive enhanced training in how to interpret risk-related medical statistics [27], which is part of this training.

One of the strengths of this study is the systematic development of the training programme on the basis of problem-based learning by staff trained in vocational education and training. Additionally, the data analysis was performed by two researchers and different data were triangulated. We documented the coding procedure transparently so that it is reproducible. However, it is important to mention some limitations of this study. First of all, not all the participants actually classified themselves as developers of health information who were able to judge the practical relevance of the training. Furthermore, the researchers who developed and conducted the training also collected and analysed the data.

## Conclusions

The results of this study will be taken into account when conducting the randomised controlled trial evaluating the implementation of the *Guideline Evidence-based Health Information*. Recruitment experiences from this pilot study indicate that recruiting institutions for the subsequent randomised controlled trial as well as corresponding training programmes might, in general, be challenging. Several aspects need to be considered: Institutions might not see the necessity for training since they consider themselves already well-trained. Currently, there are almost no incentives (e.g. from politics) for developing EBHI. On the contrary, it seems as if it is much easier to distribute health information which is not evidence-based, and the general public perceives familiar and commercial health information as being trustworthy [5]. This is problematic because well-known information is often not trustworthy and does not have a scientific basis. Moreover, the length of the training programme could be a barrier because it requires quite a large amount of time and staff resources from the participating institutions.

Training programmes and their curricula have a key role for acquiring EBM knowledge as well as the competences for developing EBHI. Theory-practice transfer and behaviour change seem to remain a major issue. It is important to develop a strategy to create incentives for health information providers to develop EBHI and improve their competences in the long term. Another challenge is that different professions are involved in developing health information, a topic which has not necessarily been addressed in their original training. It appears to be important to integrate EBM as well as the development of EBHI into the curricula of degree programmes in health sciences, since this could be a crucial occupational field for health scientists. Furthermore, structures for developing health information should be established, for instance in the context of medical guideline processes where competence and expertise is being pooled.

In addition, in Germany, a national health portal is being planned [31]. The portal is intended to optimise the search for health information and provide quality assured health information. The long-term goal is to improve the general public’s health literacy. The concept includes training opportunities for the portal’s content partners concerning the development of high-quality health information [32]. It is conceivable that the developed training programme could be offered as a continuing education opportunity in the context of the health portal in order to reach the long-term goal of improving the quality of health information.

## Supplementary information

**Additional file 1.** COREQ Checklist.

**Additional file 2.** Interview guide for the focus group interviews.

**Additional file 3.** Coding guideline.

## Data Availability

The datasets generated and analysed during the current study are available from the corresponding author on reasonable request.

## Abbreviations

COREQ: COnsolidated criteria for REporting Qualitative research
CReDECI: Criteria for Reporting the Development and Evaluation of Complex Interventions in healthcare
EBHI: Evidence-Based Health Information
EBM: Evidence-Based Medicine
RCT: Randomised Controlled Trial
