# Supplementary material for: A blended learning training programme for health information providers to enhance implementation of the Guideline Evidence-based Health Information: development and qualitative pilot study
Source: BMC Med Educ. 2020 Mar 18;20:77. doi: 10.1186/s12909-020-1966-3 (PMC7079382; doi:10.1186/s12909-020-1966-3)
Supplement: Supplementary file 1 — Additional file 1. COREQ Checklist. [file 12909_2020_1966_MOESM1_ESM.pdf]

COREQ (COnsolidated criteria for REporting Qualitative research) Checklist

Manuscript: A blended learning training programme for health information providers to enhance implementation of the *Guideline Evidence-based Health Information*: development and qualitative pilot study

No. Item	Guide questions/description	Reported on page No.
**Domain 1: Research team and reflexivity**		
Personal Characteristics		
1. Interviewer/facilitator	Which author/s conducted the interview or focus group?	5
2. Credentials	What were the researcher's credentials? E.g. PhD, MD	–
3. Occupation	What was their occupation at the time of the study?	1
4. Gender	Was the researcher male or female?	1
5. Experience and training	What experience or training did the researcher have?	5
Relationship with participants		
6. Relationship established	Was a relationship established prior to study commencement?	4
7. Participant knowledge of the interviewer	What did the participants know about the researcher? e.g. personal goals, reasons for doing the research	4
8. Interviewer characteristics	What characteristics were reported about the interviewer/facilitator? e.g. Bias, assumptions, reasons and interests in the research topic	4
**Domain 2: study design**		
Theoretical framework		
9. Methodological orientation and Theory	What methodological orientation was stated to underpin the study? e.g. grounded theory, discourse analysis, ethnography, phenomenology, content analysis	5
Participant selection		
10. Sampling	How were participants selected? e.g. purposive, convenience, consecutive, snowball	4
11. Method of approach	How were participants approached? e.g. face-to-face, telephone, mail, email	4
12. Sample size	How many participants were in the study?	5
13. Non-participation	How many people refused to participate or dropped out? Reasons?	5
Setting		
14. Setting of data collection	Where was the data collected? e.g. home, clinic, workplace	4
15. Presence of non-participants	Was anyone else present besides the participants and researchers?	5
16. Description of sample	What are the important characteristics of the sample? e.g. demographic data, date	5

Data collection		
17. Interview guide	Were questions, prompts, guides provided by the authors? Was it pilot tested?	5
18. Repeat interviews	Were repeat interviews carried out? If yes, how many?	5
19. Audio/visual recording	Did the research use audio or visual recording to collect the data?	5
20. Field notes	Were field notes made during and/or after the interview or focus group?	5
21. Duration	What was the duration of the interviews or focus group?	5
22. Data saturation	Was data saturation discussed?	5
23. Transcripts returned	Were transcripts returned to participants for comment and/or correction?	5
**Domain 3: analysis and findings**		
Data analysis		
24. Number of data coders	How many data coders coded the data?	5
25. Description of the coding tree	Did authors provide a description of the coding tree?	5
26. Derivation of themes	Were themes identified in advance or derived from the data?	5
27. Software	What software, if applicable, was used to manage the data?	5
28. Participant checking	Did participants provide feedback on the findings?	5
Reporting		
29. Quotations presented	Were participant quotations presented to illustrate the themes/findings? Was each quotation identified? e.g. participant number	6-9
30. Data and findings consistent	Was there consistency between the data presented and the findings?	6-9
31. Clarity of major themes	Were major themes clearly presented in the findings?	6-9
32. Clarity of minor themes	Is there a description of diverse cases or discussion of minor themes?	6-9

Developed from:

Tong A, Sainsbury P, Craig J: Consolidated criteria for reporting qualitative research (COREQ): a 32-item checklist for interviews and focus groups. Int J Qual Health Care 2007, 19(6):349-357.
