# Supplementary material for: A blended learning training programme for health information providers to enhance implementation of the Guideline Evidence-based Health Information: development and qualitative pilot study
Source: BMC Med Educ. 2020 Mar 18;20:77. doi: 10.1186/s12909-020-1966-3 (PMC7079382; doi:10.1186/s12909-020-1966-3)
Supplement: Supplementary file 2 — Additional file 2. Interview guide for the focus group interviews. [file 12909_2020_1966_MOESM2_ESM.pdf]

Interview guide: focus group interviews

After the first training block:

1. **First thoughts on the training days**
   - What did you like and what did you like less?
   - What suggestions for improvement do you have?
   - Did you miss something? If yes, what?
2. **Feedback on concrete teaching elements/methods**
   - Do you like the topic of the case example and do you think that it is realistic?
   - How comprehensible were the contents for you?
     - Were there any difficulties in understanding? If so, what? (e.g. terminology, work tasks, contents of the studies)
3. **How did you experience the relationship between lecture and work phases?**
4. **What expectations do you have for the online phase?**

Short feedback (flash light) before the second training block:

- **How did you cope with the online phase?**
  - How did you cope with the learning platform?
  - How did you get along with the work tasks?
  - How satisfied were you with the support during the online phase?

After the second training block:

1. **First thoughts on the training days**
   - What did you like and what did you like less?
   - What suggestions for improvement do you have?
   - Did you miss something? If yes, what?
2. **Feedback on concrete teaching elements/methods**
   - How comprehensible were the contents for you?
     - Were there any difficulties in understanding? If so, what? (e.g. terminology, work tasks, contents of the studies)

   Case example:
   - To what extent did the smoking cessation case example help you transfer the content to your own professional practice?
   - To what extent has the case example caught your interest in dealing with the topic?

5. **How did you experience the relationship between lecture and work phases?**
6. **How did you perceive the heterogeneity of the participants (e.g. different professions) within the group?**
7. **To what extent can you imagine applying what you have learned in your professional practice after this training?**
   - If yes, what and how?
   - If not, why?
