# Supplementary material for: A blended learning training programme for health information providers to enhance implementation of the Guideline Evidence-based Health Information: development and qualitative pilot study
Source: BMC Med Educ. 2020 Mar 18;20:77. doi: 10.1186/s12909-020-1966-3 (PMC7079382; doi:10.1186/s12909-020-1966-3)
Supplement: Supplementary file 3 — Additional file 3. Coding guideline. [file 12909_2020_1966_MOESM3_ESM.pdf]

Coding guideline: qualitative content analysis

Category	Definition	Coding rules	Anchor examples
**Framework conditions of the training**	**Contextual factors**	Coding unit: Clear meaning component (seme) in the text	
Heterogeneity of the target group and adequacy of the training for the target group	Heterogeneity of the target group regarding their skills and prior knowledge, adequacy of the training for the target group		*“I think it was bit by bit…actually. One was gradually introduced. I’m a beginner. Not from the EBM department. Therefore, I found it to be a good introduction to the topic.”* *“Concerning the target group, I was in-between. I had some previous knowledge but not really EBM knowledge. I could follow very well. Statistical basics are present. I didn’t have to think much, but the stumbling block of EBM became clear.”*
Time frame	Adequacy of the time frame (timing and duration of the training)		*“The time was going by fast. It was very compact.”*
Online phase: learning management system ILIAS	Technical realisation of the online phase		*“Concerning the learning management system. I think it couldn’t be operated intuitively. A clear layout would be better. Also a bit better structured.”*
**Interaction**	**Exchange between the involved people**	Coding unit: Clear meaning component (seme) in the text	
Interaction between learners and teachers	Mutual interaction of learners and teachers regarding their actions and communication		*“Overall, I found the atmosphere to be very pleasant. I got the impression that everyone can ask questions.”*
Team teaching	Teacher cooperation, simultaneous teaching by two teachers		*“How you communicate, so enthusiastically. You want it. Without rebuke. Excellent. That’s very appealing.”*

Category	Definition	Coding rules	Anchor examples
Relationship between lecture and work phases	Balance between lecture and work phases		*"The relationship between lecture and work phases turned out well."*
**Methods**	**Use of teaching methods**	Coding unit: Clear meaning component (seme) in the text	
Adequacy of methods regarding teaching and learning goals	Contribution to achieving the teaching and learning goals		*"The practical exercises were very helpful to get a deeper understanding."*
Realisation of the teaching methods	Successful implementation of the teaching methods (e.g. group work)		*"Towards the end, [the work task on] user testing… I think there was a bit too much switching. Group building could be simplified."*
Acceptance of the teaching methods	Acceptance of the teaching methods by the learners		*"75 minutes is quite long. It was inconvenient doing it [the work task] as the first thing in the morning. In the morning, listening is more convenient."*
**Planning of the training programme**	**Smooth running of teaching/adequacy of the training programme**	Coding unit: Clear meaning component (seme) in the text	
Schedule	Time constraints in planning and their feasibility	Were the planned time slots realistic? Time frame (under framework conditions) refers to the timing and duration of the whole training.	*"I think I've done everything. But I'm much faster than my colleagues who've never done that before. And some of us had completed the first work task in a large group. […] Self-organisation isn't as easy sometimes. But we did a quite good job."*
Common thread	Structuring of the lessons, transparency of the teaching process		*"What was missing… The big picture of the whole training concept. What are we doing how, and what is building on what and where can I expect what. Because some things will be part of the third attendance day, and I don't know, will they be a topic or do I have to ask."*

Category	Definition	Coding rules	Anchor examples
Transparency of teaching and learning goals	Clarity/transparency of teaching and learning goals for the learners		*“One should think about the goal, what should be remembered? What’s the teaching goal? And then think about the question ‘How to teach different groups with different previous knowledge?’.”*
**Value and design of the learning and teaching materials**	**Usability of the materials**	Coding unit: Clear meaning component (seme) in the text	
Practical relevance and value	Feedback on the learning and teaching materials: use of the materials in practice (e.g. folders, printed presentations)		*“I liked the real texts. The use of real studies. That’s very concrete. That’s the real work and the right material to work on. It’s irritating if it [the studies in the work tasks] looks completely different.”*<br>*“I’m glad to have this folder, because I’ve the feeling that I have got to look up quite a lot.”*
Design	Comprehensibility, design and logical structure of the learning and teaching materials		*“If the pages had been numbered consecutively, it would have been helpful.”*
Adequacy for the target group	Adequacy of the learning and teaching materials for the target group		*“The real texts. One could offer the studies in original language depending on who is attending the training.”*
**Comprehensibility**	**Comprehensibility of the contents of the training**	Coding unit: Clear meaning component (seme) in the text	
Studies	Comprehensibility of the study characteristics and contents		*“I was sometimes overwhelmed by the work tasks. […] Especially, the critical appraisal of the review. That overwhelmed me a bit.”*
Online tasks	Comprehensibility of the online tasks		*“If you had left me alone with this work task [on systematic literature search], I would have failed.”*
Terminology	Comprehensibility of terms		*“[…] about the comprehensibility of terms or deeper understanding. Further explanations would be necessary. It could be a good thing for the handout. A glossary.”*

Category	Definition	Coding rules	Anchor examples
Criteria of evidence-based health information (EBHI)	Comprehensibility of the EBHI criteria		*"Especially the goal and target group, that has to be focused very clearly before developing information or it has to be taken into consideration. Those are aspects that weren't clear to me before. Even if I've read it. I completely lost sight of it. Because it's logical that we do it to…? But why are we doing it? I found it quite interesting."*
Missing contents and examples	Lack of input, missing examples that contribute to comprehension		*"I think it's important to discuss the interface between communication topics and EBM."*
**Practical relevance and feasibility**	**Applicability in practice**	Coding unit: Clear meaning component (seme) in the text	
Acceptance of the contents	To what extent can the participants accept the contents of the training?		*"It was much more comprehensible today than the two days before. This was mainly because of the practical examples, which are more familiar to me from daily business. I liked it really a lot."*
Case example	Did the case example arouse interest?, realism of the case example, assistance for transfer to own practice		*"The example and the topic were well chosen because it's a topic everyone is interested in and that everyone knows."*
Theory-practice transfer	Transfer/application of the acquired knowledge/competencies in practice		*"Would I dare to develop an EBHI based on those three days of training? No way. I have to say so. I've great respect. But I think one sees those things with different eyes. And details are revealed, critical aspects, and that helps."*
Required institutional resources	Resources that must be available in the institutions to implement the guideline		*"We have the luxury to have a department for everything. There are no barriers."*
Chances	Reasons that underline the implementation of the learning content in practice		*"The training definitely confirmed the methods of our institution and it showed that the way we work complies with the suggested gold standard."*
